# Inhibition of pancreatic cancer stem cell characteristics by α‐Mangostin: Molecular mechanisms involving Sonic hedgehog and Nanog

**DOI:** 10.1111/jcmm.14178

**Published:** 2019-02-03

**Authors:** Yiming Ma, Wei Yu, Anju Shrivastava, Rakesh K. Srivastava, Sharmila Shankar

**Affiliations:** ^1^ Kansas City VA Medical Center Kansas City Missouri; ^2^ Department of Oncology St. Joseph's Hospital and Medical Center Phoenix Arizona; ^3^ Department of Pharmaceutical Sciences University of Missouri‐Kansas City Kansas City Missouri; ^4^ Department of Pathology, School of Medicine University of Missouri‐Kansas City Kansas City Missouri; ^5^ Present address: Southeast Louisiana Veterans Health Care System New Orleans Louisiana; ^6^ Present address: Department of Genetics Stanley S. Scott Cancer Center, Louisiana State University Health Sciences Center New Orleans Louisiana

**Keywords:** cancer stem cells, chromatin immunoprecipitation, Nanog, pancreatic cancer, Sonic hedgehog, α‐Mangostin

## Abstract

The current investigation was intended to elucidate the molecular mechanism of α‐Mangostin in the regulation of pancreatic cancer stem cell (CSC) characteristics. Here, we demonstrate that α‐Mangostin inhibited cell proliferation in pancreatic CSCs and cancer cell lines while it showed no effect on human pancreatic normal ductal epithelial cells. Also, α‐Mangostin inhibited colony formation and induced apoptosis in these cells. Further, α‐Mangostin inhibited the self‐renewal capacity of CSCs isolated from human primary tumours and Kras^G12D^ mice. Furthermore, α‐Mangostin inhibited the invasive and metastatic ability of pancreatic CSCs by suppressing the epithelial‐to‐mesenchymal transition (EMT) via up‐regulation of E‐cadherin and down‐regulation of mesenchymal phenotype by inhibiting N‐cadherin, Snail and Slug expression. Interestingly, the pluripotency maintaining factors and CSC markers were inhibited by α‐Mangostin thus suggesting that α‐Mangostin can target CSCs to inhibit pancreatic cancer effectively. Gli signalling plays a crucial role in the self‐renewal and pluripotency of CSCs. α‐Mangostin inhibited the Gli transcription and the expression of Gli target genes (Nanog, Oct4, c‐Myc, Sox‐2 and KLF4) in CSCs. Using ChIP assay, we demonstrated that Nanog could directly bind to promoters of Cdk2, Cdk6, FGF4, c‐Myc and α‐Mangostin inhibited Nanog binding to these promoters. Conversely, the inhibitory effects of the α‐Mangostin on CSC proliferation and Gli or Nanog transcription and their targets were abrogated by either enforced activation of sonic hedgehog (Shh) or by the overexpression of Nanog. Taken together, our studies suggest that α‐Mangostin may act as Gli inhibitor and establishes the pre‐clinical significance of α‐Mangostin for the prevention and treatment of pancreatic cancer.

## 1 INTRODUCTION

Pancreatic cancer is a devastating disease and is the fourth most common cause of cancer‐related mortality in the United States.^1^ Pancreatic cancer exhibits the poorest prognosis from all other cancers, and the overall 5‐year survival rate continues to be less than 6%.^2^, ^3^ Pancreatic cancer is characterized by slow growth, late detection and resistance to chemotherapy and radiation and is associated with high mortality rates even after surgery.^3^ Unfortunately, by the time the disease is diagnosed most of the pancreatic cancer patients present with unresectable advanced malignancy due to early metastasis. Accumulating evidence supports the role of cancer stem cells (CSCs) in cancer initiation, progression, metastasis and chemotherapy failure.^4^ Despite increased advancement in our understanding of the disease progression, diagnosis and therapeutics, available treatment options are limited. Chemo‐ and radio‐therapies have been largely ineffective and associated with enhanced drug toxicity, drug resistance and frequent redevelopment of the metastatic disease. Further, poor bioavailability of the drug and undesirable side effects are other significant limitations in the effective management of pancreatic cancer. Thus, it is highly desirable to develop an increased understanding of the pathogenesis of the disease, for effective disease management and the development of effective strategies by non‐toxic natural agents for the prevention and treatment of pancreatic cancer.

Hedgehog (Hh) signalling pathway is crucially involved in vertebrate development.^5^ It is inactive in mature cells of normal adult and found to be aberrantly hyper‐activated in pancreatic cancer and various other malignancies. Evidence suggests that Hh signalling can regulate tissue homeostasis by controlling the production of stem or progenitor cells.^6^ Deregulation of the Hh signalling pathway is associated with various malignancies. In several of our recent reports, we have demonstrated that for the prevention of pancreatic cancer, a variety of natural products and small molecules displayed antiproliferative properties through targeting the sonic hedgehog (Shh) signalling pathway.^7^, ^8^, ^9^, ^10^, ^11^, ^12^ Upon binding of the Shh ligand to transmembrane Patched (Ptch) receptor results in the withdrawal of inhibitory effects of Patched on smoothened.^5^ Thus, the pathway is activated via smoothened through Hh protein stimulation or by the loss of patched activity through Ptch mutations. Activation of the Hh pathway via smoothened induces Gli transcriptional activity.

Several reports have demonstrated that Hh pathway activation induces stem cell markers and is involved in the enhancement of epithelial‐to‐mesenchymal transition (EMT) thus regulating metastasis in various malignancies including pancreatic ductal adenocarcinoma.^5^, ^13^ The involvement of Shh increases dramatically from PanIN lesions to PDAC to metastatic tumours.^14^ Therefore, targeting the Shh pathway is regarded as a beneficial strategy for the prevention and treatment of pancreatic cancer.

Wnt, Shh and several other extrinsic developmental pathways play significant roles in the maintenance and regulation of pluripotency and self‐renewal capacity of progenitor and stem cells.^15^ Nanog is a transcription factor which is one of the crucial downstream effectors of these signalling pathways and also a direct transcriptional target of Gli.^16^, ^17^ Nanog modulates pluripotency, maintain self‐renewal and block differentiation.^18^ Nanog is expressed highly in germline stem cells, tumours, carcinomas and seminomas.^19^, ^20^ In addition to Gli, various other transcription factors such as Oct4, FoxD3 and P53 can regulate Nanog transcription.^21^, ^22^ Interestingly, Nanog can cooperate with Oct4 and Sox2 in maintaining self‐renewal capacity and pluripotency in stem cells.^23^ As Nanog is a direct target of the Shh pathway, we will examine the regulation of Shh‐Nanog pathway by α‐Mangostin in pancreatic CSCs.

As natural product‐based compounds are non‐toxic, can target multiple pathways and can negatively impact the self‐renewal capacity of CSCs, they can be developed as an attractive strategy for the prevention and treatment of pancreatic cancer. Mangosteen plant (Garcinia mangostana) grows abundantly in Southeast Asia mainly in the Sunda Islands and the Moluccas of Indonesia and other parts of tropical South America.^24^, ^25^, ^26^ Mangosteen is rich in xanthonoid and phytochemicals. α‐Mangostin is a xanthonoid derived from Mangosteen which is well tolerated and safe. It possesses antioxidant, anticancer and anti‐inflammatory properties that are highly relevant to humans.^24^, ^25^, ^27^, ^28^, ^29^, ^30^ However, the underlying molecular mechanisms of α‐Mangostin in the inhibition of pancreatic cancer by targeting CSCs and Shh‐Nanog pathway are not well understood. Therefore, α‐Mangostin holds great promise and can be developed as an anticancer agent.

The objective of this study was to delineate the molecular mechanism of α‐Mangostin in the regulation of pancreatic CSC characteristics. Our data demonstrate that α‐Mangostin inhibits cell proliferation in pancreatic CSCs and cancer cell lines. α‐Mangostin is non‐toxic to human pancreatic normal ductal epithelial cells. Also, α‐Mangostin inhibits the colony formation and induces apoptosis selectively in pancreatic CSCs and cancer cell lines, compared to the pancreatic normal ductal epithelial cells. Further, the self‐renewal capacity of CSCs isolated from human primary tumours and Kras^G12D^ mice was inhibited by α‐Mangostin.

Furthermore, α‐Mangostin inhibited the invasive and metastatic ability of pancreatic CSCs by modulation of cadherin expression and suppressing the transcription factors Snail, and Slug, which regulates the EMT. α‐Mangostin inhibited the expression of Nanog and pluripotency promoting factors (Oct4, c‐Myc, Klf4 and Sox2) in CSCs. Using ChIP assay, we demonstrate that Nanog can directly bind to promoters of Cdk2, Cdk6, FGF4, c‐Myc and this Nanog binding was inhibited by α‐Mangostin. Interestingly, α‐Mangostin inhibits Gli transcription and expression. Conversely, the inhibitory effects of α‐Mangostin on pancreatic CSC proliferation and Gli or Nanog transcription and their targets was abrogated by either enforced activation of Shh or by the overexpression of Nanog. Taken together, these results suggest that α‐Mangostin may act as Gli inhibitor and also establishes the pre‐clinical significance of α‐Mangostin for the prevention and treatment of pancreatic cancer.

## 2 MATERIALS AND METHODS

### 2.1 Reagents

CD24, CD44, CD133, Nanog, Oct4, Sox2, KLF4, c‐Myc, Gli1, Gli2, Patched1, Patched2, Smoothened, Bcl‐2, Cyclin D1, E‐cadherin, N‐cadherin, Snail, Slug and Nanog antibodies were obtained from Cell Signaling Technology (Danvers, MA). Shh protein and anti‐β‐actin antibody were purchased from Santa Cruz Biotechnology Inc (Santa Cruz, CA). α‐Mangostin (98% pure) was obtained from the LKT (St. Paul, MN). Accutase was purchased from Innovative Cell Technologies, Inc (San Diego, CA). Matrigel was purchased from BD Bioscience (San Jose, CA). Crystal violet was purchased from Sigma‐Aldrich (St. Louis, MO). TRIZOL was purchased from Invitrogen (Grand Island, NY). Luciferase assay kit was purchased from Promega.

### 2.2 Cell culture

Pancreatic cancer cell lines AsPC‐1 and PANC‐1 were used, and these cells were purchased from American Type Culture Collection (Manassas, Virginia) purchase. Cells were grown and frozen in liquid nitrogen for future use. Cells from second and third passages were used for the experiments. ATCC utilizes short tandem repeat (STR) profiling to authenticate the cell lines. PANC‐1 possesses mutations in p53 and K‐ras genes in codon 273 and codon 12 respectively. AsPC‐1 harbours mutation on codon 12 of K‐ras gene. CD133+/CD44+/CD24+/ESA+human pancreatic CSCs were isolated from primary tumours as described previously.^26^ Pancreatic CSCs isolation and characterization from KrasG12D mice were performed as described elsewhere.^26^ Pancreatic CSCs were grown in specialized growth medium (Celprogen, Inc, Torrance, California) which contained 1% N2 Supplement (Invitrogen), 2% B27 Supplement (Invitrogen), 20 ng/mL human platelet growth factor (Sigma‐Aldrich), 100 ng/mL epidermal growth factor (Invitrogen) and 1% antibiotic‐antimycotic (Invitrogen). Pancreatic CSCs and cancer cell lines were cultured at 37°C in a humidified atmosphere of 95% air and 5% CO_2_.

### 2.3 Cell proliferation and apoptosis assays

Pancreatic cancer cells and CSCs (1.5 × 10^4^) in 1 mL of culture medium were incubated for 24 to 48 hours time‐points with various concentrations of 0‐10 µmol/L of α‐Mangostin. Cell viability and apoptosis were measured by trypan blue assay using Countess™ Automated Cell Counter (Invitrogen) and TUNEL assay respectively.^7^

### 2.4 Colony formation assay

For colony formation assay, pancreatic cancer cells and CSCs were seeded at a low density into 6‐well plates and then treated with or without α‐Mangostin for up to 2 weeks. Cell culture medium containing either α‐Mangostin or DMSO was renewed every 3 days. After fixation of the colonies with cold methanol, 0.5% crystal violet was used to stain them. The colonies were imaged with a microscope, and the number of colonies was counted.

### 2.5 Spheroid assay

We performed spheroid formation assays as described elsewhere.^7^ Briefly, cells at 100‐500 cells/mL density were plated in ultra‐low attachment plates. The spheroid formation in suspension was evaluated under a microscope after 10 days of culture.

### 2.6 Motility, transwell migration and invasion assays

Assays for cell motility, transwell migration and invasion have been performed as we described elsewhere.^7^, ^8^, ^26^

### 2.7 Gene expression by quantitative RT‐PCR analysis

Total RNA in cells was isolated using TRIzol reagent (Invitrogen). About 2 μg of the extracted RNA was reverse transcribed into cDNA using High‐Capacity cDNA Reverse Transcription Kit (Thermo Fisher Scientific). qRT‐PCR was carried out using fast SYBR Green Master Mix (Thermo Fisher Scientific). Relative mRNA expressions were compared with controls and evaluated using 2^−ΔΔCt^ methods.

### 2.8 Western blot analysis

Cells were lysed in RIPA buffer and sonicated. The extracts were further centrifuged, and the supernatant was collected and stored at −80°C for future experiment and Western blot analysis. iMark (BIO‐RAD) protein assay was used to quantitate the cell lysates. 40‐50 μg of protein lysate was loaded on 10% or 12% bis‐acrylamide gels for protein separation and transferred onto PVDF membranes. The blot was blocked with 5% nonfat dry milk in TBS‐T (TBS and 0.01% Tween‐20) buffer and incubated with various primary antibodies in TBS‐T overnight at 4°C. The immunoblots were washed thrice with TBS‐T and incubated with secondary antibody (1:20 000). For chemiluminescence reactions, Super Signal West Pico substrate (Thermo Fisher, Waltham, MA) was used as per the manufacturers' protocol. For reuse, the blots were washed in stripping buffer for 30‐60 minutes at room temperature.

### 2.9 Chromatin immunoprecipitation (ChIP)

Pancreatic CSCs were treated with various doses of α‐Mangostin, crosslinked and sonicated. The crosslinked sheared chromatin samples were then incubated with 3 μg of Nanog antibody and 5 μL of protein‐A and protein‐G magnetic beads. The ChIP DNA was further purified, and 1‐3 µL elutions were used to measure enrichment using quantitative real‐time PCR. ChIP‐derived DNA was electrophoresed on 2% agarose gels.

### 2.10 Gli and Nanog reporter assay

Measurement of Gli and Nanog reporter activities was performed as we described elsewhere.^7^, ^31^ In brief, pancreatic cancer cells were stably transduced with lentiviral particles expressing cop‐GFP and luciferase genes (pGreen Fire1‐4xGli or Nanog‐mCMV‐EF1‐Neo). The cells for transcription assay were seeded in 96‐well plates and treated with or without α‐Mangostin (0‐10 µmol/L) for various time‐points. According to the manufacturer's instructions (Promega Corp., Madison, WI), luciferase reporter activity was measured at the end of incubation period.

### 2.11 Statistical analysis

The results presented are representative of three independent experiments run in triplicate, unless otherwise indicated. Student *t* test or ANOVA was used to analyse the differences between groups. Differences among groups were considered significant at *P* < 0.05.

## 3 RESULTS

### 3.1 α‐Mangostin negatively impacts on cell proliferation and is associated with the induction of apoptosis in pancreatic CSCs and cell lines and has no effect on immortalized human pancreatic normal ductal epithelial cells

**Figure 1:**
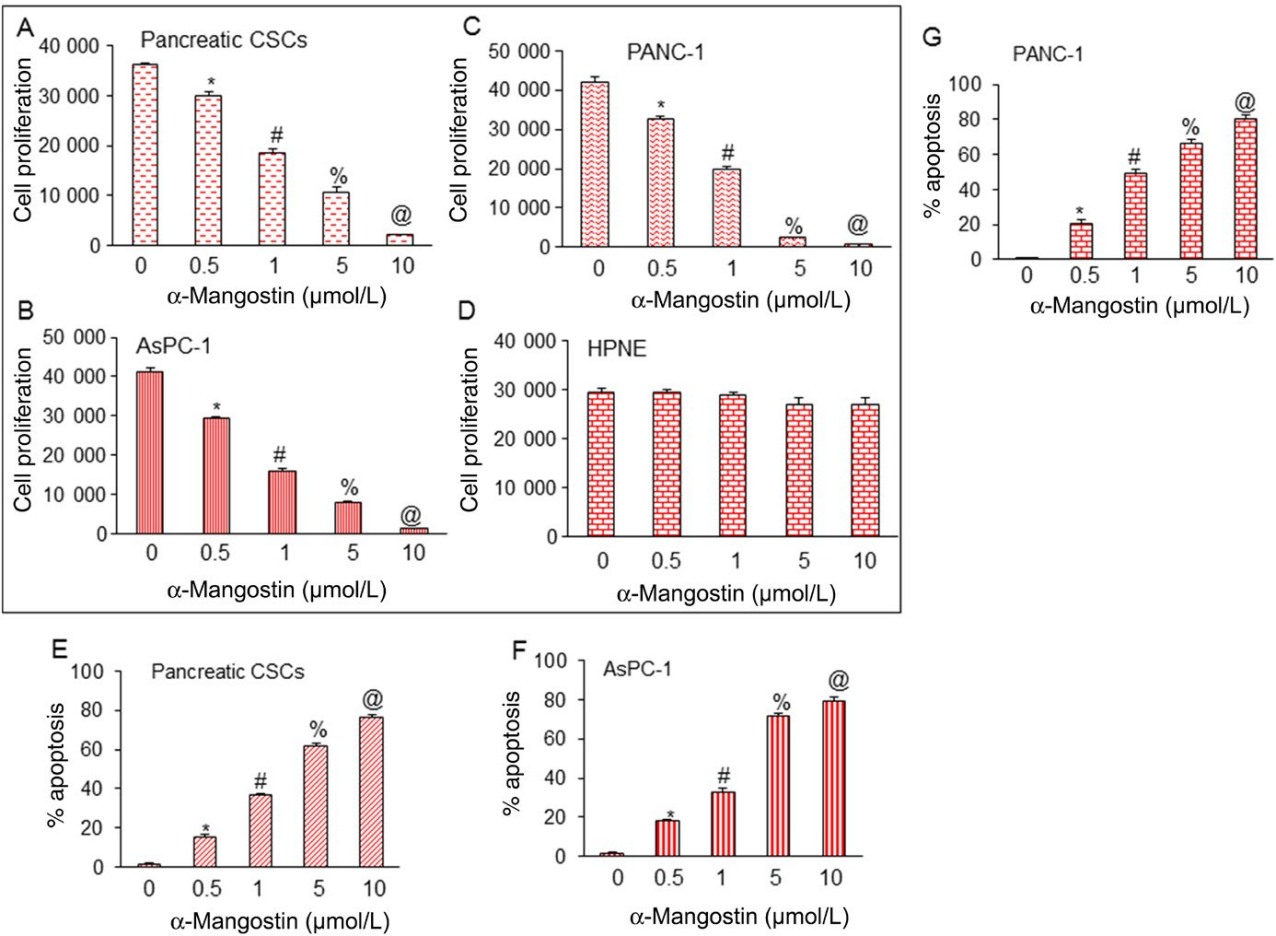
α‐Mangostin inhibits cell proliferation and induces apoptosis in pancreatic cancer stem cells (CSCs) and cell lines but has no effect on human pancreatic normal ductal epithelial (HPNE) cells. (A‐D), CD44+CD24+ESA+CSCs were isolated from human primary pancreatic tumours. Pancreatic CSCs, cancer cell lines (AsPC‐1 and PANC‐1) and (HPNE) cells were treated with α‐Mangostin (0‐10 µmol/L) for 48 h, and cell proliferation was measured by trypan blue assay. (E‐G), Pancreatic CSCs and cancer cell lines (AsPC‐1 and PANC‐1) were treated with α‐Mangostin (0‐10 µmol/L) for 48 h, and apoptosis was measured by TUNEL assay. Data represent mean (n = 4) ± SD. *, #, % and @ = significantly different from control, and each other, *P* < 0.05

Unregulated growth of cancer cells is a hallmark of several solid tumours including pancreatic cancer. We, therefore, focused on examining the effects of α‐Mangostin on cell proliferation. Pancreatic cancer stem cells (CSCs), cell lines (AsPC‐1 and PANC‐1) and human pancreatic normal ductal epithelial cells (HPNE) were exposed to various concentrations of α‐Mangostin. As demonstrated in (Figure 1A‐D), α‐Mangostin inhibited cell proliferation of pancreatic CSCs and cell lines in a dose‐dependent manner whereas α‐Mangostin did not affect the cell viability of HPNE cells. Taken together, these data suggest that α‐Mangostin can be used to inhibit the growth of pancreatic CSCs and cell lines, as interestingly it exerted no toxicity to the normal HPNE cells.

As α‐Mangostin inhibited cell proliferation in pancreatic cancer cells, we proposed to investigate further if it could regulate apoptosis in these cells. Interestingly, α‐Mangostin treatment lead to a dose‐dependent induction of apoptosis in pancreatic CSCs and cell lines as demonstrated in (Figure 1E‐G). In contrast, no significant apoptosis was observed in α‐Mangostin‐treated HPNE cells (data not shown). These data, therefore, suggest that α‐Mangostin negatively impacts on cell proliferation and is associated with the induction of apoptosis in pancreatic CSCs and cell lines and is non‐toxic to normal pancreatic epithelial cells. Therefore, it may have the potential to be used in the prevention and treatment of pancreatic cancer.

### 3.2 α‐Mangostin targets the pancreatic CSCs self‐renewal capacity, stemness and inhibits spheroid and colony formation

**Figure 2:**
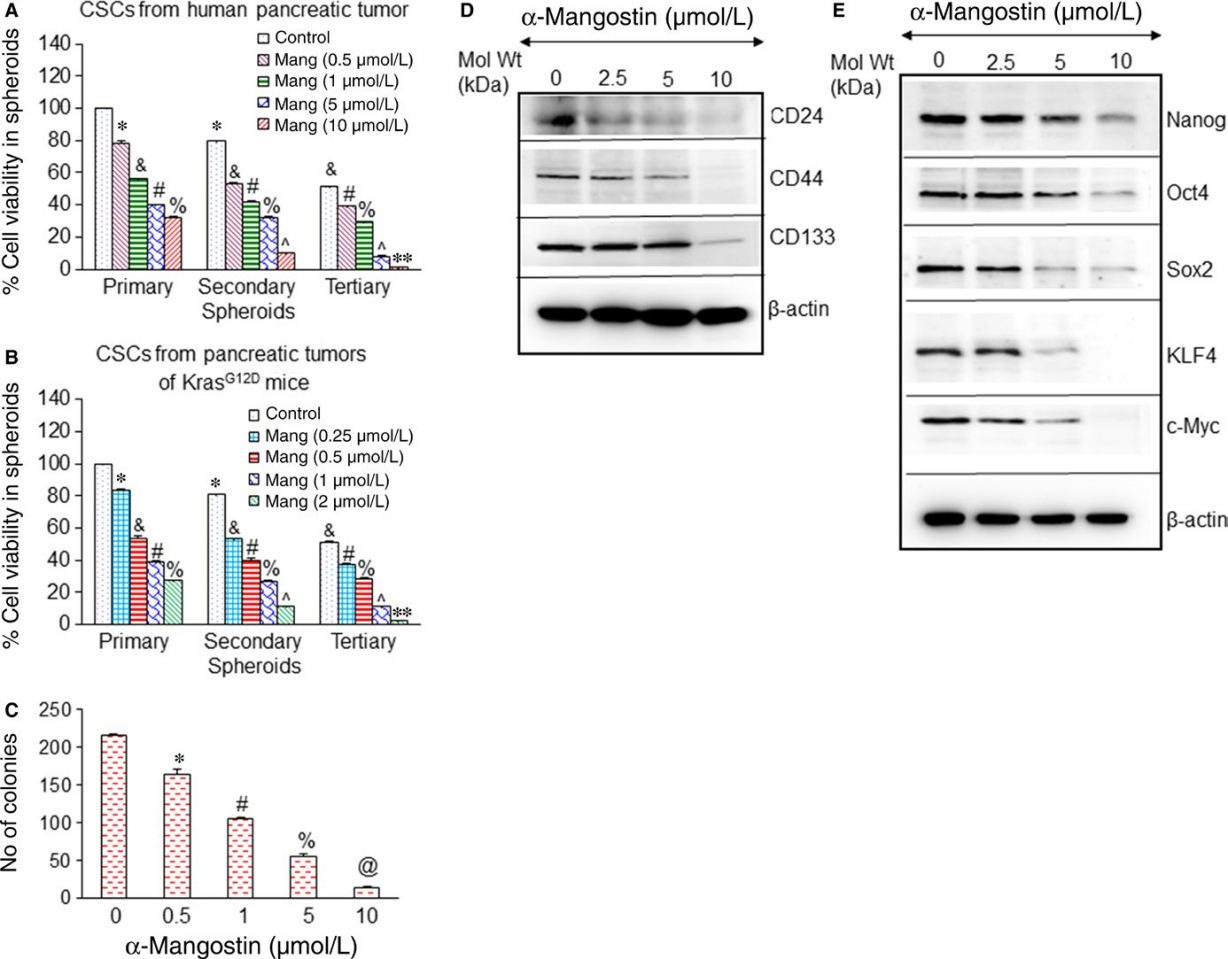
α‐Mangostin inhibits cell viability in spheroids, and colony formation and the expression of stem cell markers and pluripotency maintain factors by CSCs from pancreatic tumours. (A and B), CSCs were isolated from pancreatic tumours of humans and KrasG12D mice and treated with α‐Mangostin (0‐10 µmol/L) for 7 d to obtain primary spheroids. At the end of the incubation period for a week, the spheroids were collected, reseeded and treated with α‐Mangostin for another week to obtain secondary spheroids. Further, secondary spheroids were collected, reseeded and treated with α‐Mangostin for another week to obtain tertiary spheroids. Cell viability in the spheroids was measured by trypan blue assay at the end of 7, 14 and 21 d. Data represent mean ± SD. *, &, #, %, ˄ and ** = significantly different from control, *P* < 0.05. C, In soft agar, human pancreatic CSCs were seeded and treated with α‐Mangostin (0‐10 μmol/L) for 21 d. At the end of the incubation period, the number of colonies was counted. *, #, % and @ = significantly different from control (n = 4), *P* < 0.05. D, Human pancreatic CSCs were treated with α‐Mangostin (0‐10 μmol/L) for 48 h. The expression of CD24, CD44 and CD133 was measured by the Western blot analysis. E, human pancreatic CSCs were treated with α‐Mangostin (0‐10 μmol/L) for 48 h, and the expression of Nanog, Oct4, Sox2, KLF4 and c‐Myc was measured by the Western blot analysis. β‐actin was used as a loading control

A significant characteristic of CSC is its ability to form spheroids in suspension which is reflective of its capability of stemness, reconstitution and propagation of tumours.^8^, ^32^ Thus, to examine the effect of α‐Mangostin on the growth of CSCs isolated from primary pancreatic tumours from human and KrasG12D mice, we measured spheroid formation abilities in the presence and absence of α‐Mangostin. As shown in Figure 2A,B, in CSCs isolated from pancreatic tumours of human and KrasG12D mice, the cell viability of primary, secondary and tertiary spheroids was inhibited by α‐Mangostin. In the α‐Mangostin‐treated groups, smaller and fewer spheroids were formed than that in the control group (data not shown). Further, α‐Mangostin impaired the colony formation ability of human pancreatic CSCs in a dose‐dependent manner (Figure 2C). We, therefore, examined the underlying mechanism for these inhibitory effects of α‐Mangostin on the regenerative and survival capacity of human pancreatic CSCs. α‐Mangostin inhibited the expression of pancreatic CSC markers CD24, CD44 and CD133 (Figure 2D). Thus, the inhibition of pancreatic CSC markers by α‐Mangostin indicates that it can hinder the CSC population.

Pluripotency maintaining factors play a critical role in maintaining stemness and are highly expressed in CSCs. We therefore next examined the effects of α‐Mangostin on the expression of pluripotency maintaining factors in human pancreatic CSCs. As demonstrated in Figure 2E, α‐Mangostin inhibited the expression of Nanog, Oct4, Sox2, KLF4 and c‐Myc, suggesting that α‐Mangostin is capable of inhibiting the self‐renewal capacity of CSCs. Taken together, these results strongly suggest that as α‐Mangostin can inhibit spheroids, colony formation, stem cell marker and pluripotency maintaining factor expression, it can be potentially used, for targeting pancreatic CSCs.

### 3.3 Inhibitory effects of α‐Mangostin on Shh signalling pathway and Gli transcriptional targets

**Figure 3:**
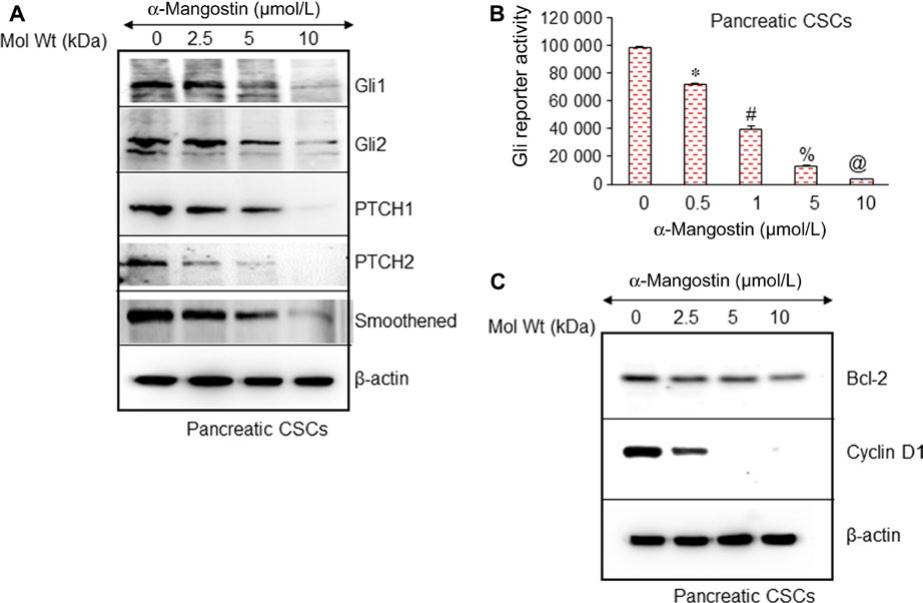
α‐Mangostin inhibits the components of the sonic hedgehog (Shh) pathway, Gli transcription and markers of cell proliferation and cell cycle. A, Pancreatic CSCs were treated with α‐Mangostin (0‐10 µmol/L) for 48 h. The expression of Gli1, Gl2, Patched‐1, Patched‐2 and smoothened was measured by the Western blot analysis. β‐actin was used as a loading control. B, Gli‐responsive GFP/firefly luciferase viral particles were used to transduce pancreatic CSCs (pGreen Fire1‐Gli with EF1, System Biosciences). After transduction, the culture medium was replaced, and CSCs were treated with α‐Mangostin (0‐10 µmol/L) for 24 h. Gli reporter activity was measured as we described elsewhere.^16^ *, #, % and @ = significantly different from control, and each other, *P* < 0.05. C, α‐Mangostin inhibits the expression of Bcl‐2 and cyclin D1. Pancreatic CSCs were treated with α‐Mangostin (0‐10 µmol/L) for 48 h, and the expression of Bcl‐2 and cyclin D1 was measured by the Western blot analysis. β‐actin was used as a loading control

We have demonstrated that Shh signalling pathway is highly activated in pancreatic cancer.^7^, ^11^, ^33^ We examined the expression of Shh pathway components to analyse the effects of α‐Mangostin in pancreatic CSCs. Gli1, Gli2, Patched1, Patched2 and smoothened protein expression was inhibited by α‐Mangostin as measured using Western blot analysis (Figure 3A). As Gli can regulate its own expression as well as the expression of Patched as they are both its direct transcriptional targets. To examine the effects of α‐Mangostin, we next measured the Gli transcriptional activity using luciferase reporter assay. As demonstrated in (Figure 3B), Gli reporter activity was inhibited in a dose‐dependent manner by α‐Mangostin. These data suggest that α‐Mangostin inhibits survival of pancreatic cancer cells by facilitating the inhibition of Shh pathway components and Gli target proteins.

Cell proliferation and cell cycle play crucial roles in maintaining the CSC population, we thus measured the expression of Bcl‐2 and Cyclin D1 (Figure 3C). Cyclin D1 acts at the G1/S phase of the cell cycle. α‐Mangostin inhibited Bcl‐2 and Cyclin D1 protein expression suggesting that α‐Mangostin can inhibit cell proliferation and cell cycle and induce apoptosis by regulating these critical factors.

### 3.4 α‐Mangostin inhibits binding of Nanog to its target genes (Cdk2, Cdk6, FGF4, c‐Myc and Oct4) and Nanog transcription

**Figure 4:**
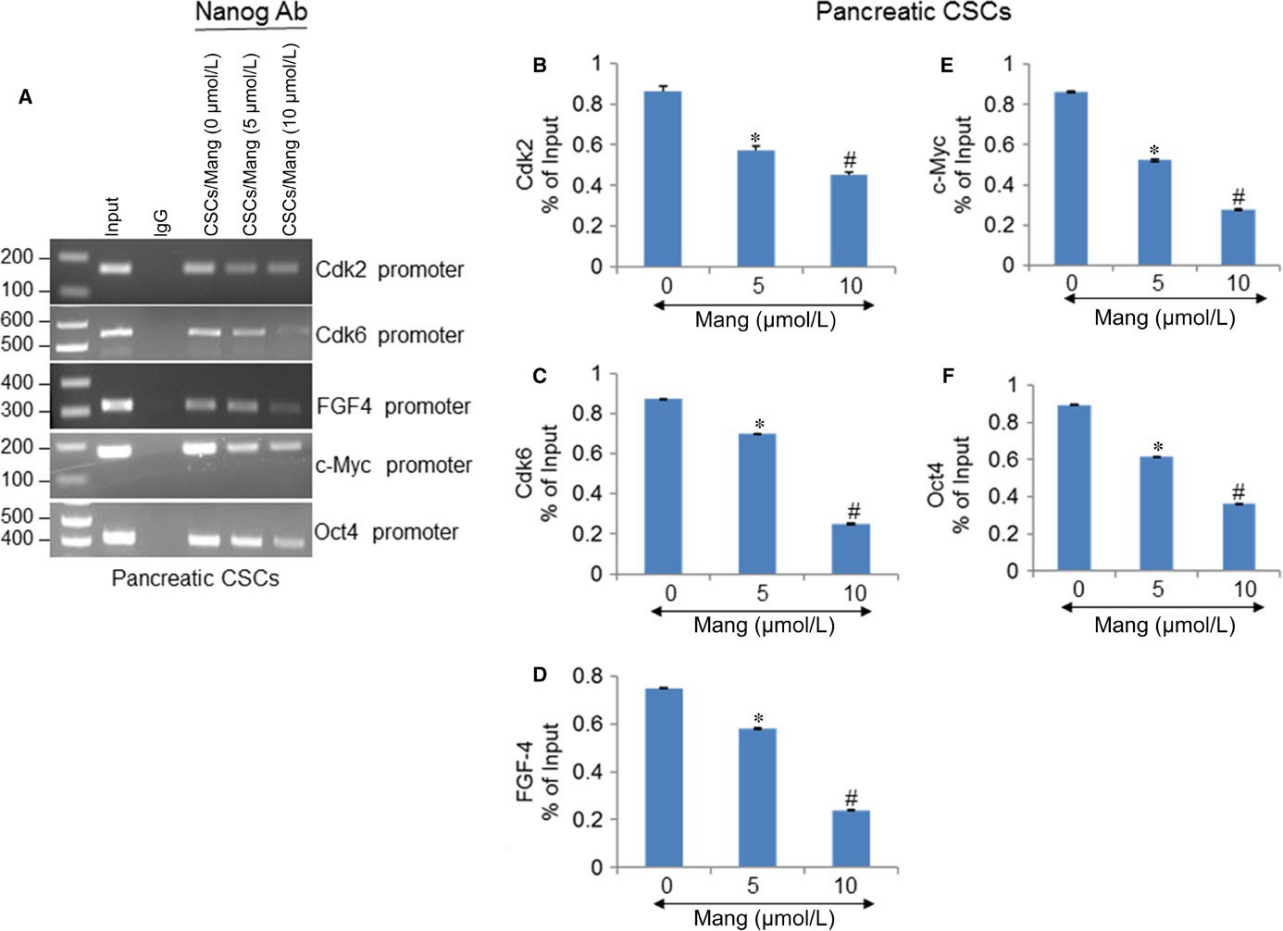
α‐Mangostin inhibits binding of Nanog to its target genes (Cdk2, Cdk6, FGF4, c‐Myc and Oct4). A, Pancreatic CSCs were treated with α‐Mangostin (0‐10 µmol/L) for 24 h. Cells were harvested, and chromatin immunoprecipitation assays were performed with the anti‐Nanog antibody as described in Materials and Methods. PCR was performed to examine the binding of Nanog to Cdk2, Cdk6, FGF4, c‐Myc and Oct4 promoters. Lane 1 = input, Lane 2 = immunoprecipitation (IP) with an anti‐IgG antibody, Lanes 3‐5 = IP with the anti‐Nanog antibody of cell lysates from CSCs treated with 0, 5 or 10 µmol/L α‐Mangostin respectively. (B‐F), Nuclear extracts were prepared, and chromatin immunoprecipitation assays were performed as described above. qRT‐PCR was used to examine the binding of Nanog to Cdk2, Cdk6, FGF4, c‐Myc and Oct4 promoters. Data represent mean (n = 4) ± SD. *, and # = significantly different from control, and each other, *P* < 0.05

In the maintenance of self‐renewal and pluripotency, Nanog is considered to play a critical role. We have demonstrated increased levels of Nanog expression in pancreatic CSCs and cell lines. As Nanog is a transcription factor, the effects of α‐Mangostin on Nanog binding to the promoters of its target genes were examined. We performed chromatin immunoassays for investigating the binding of Nanog to promoters of Cdk2, Cdk6, FGF4, c‐Myc and Oct4 in the presence and absence of α‐Mangostin. As shown by ChIP‐PCR assay in Figure 4A, Nanog can bind to Cdk2, Cdk6, FGF4, c‐Myc and Oct‐4 target gene promoters. However, the binding of Nanog to these promoters was significantly inhibited by α‐Mangostin. We confirmed these ChIP‐PCR data with qRT‐PCR where α‐Mangostin inhibited the binding of Nanog to Cdk2, Cdk6, FGF4, c‐Myc and Oct4 genes (Figure 4B‐F).

**Figure 5:**
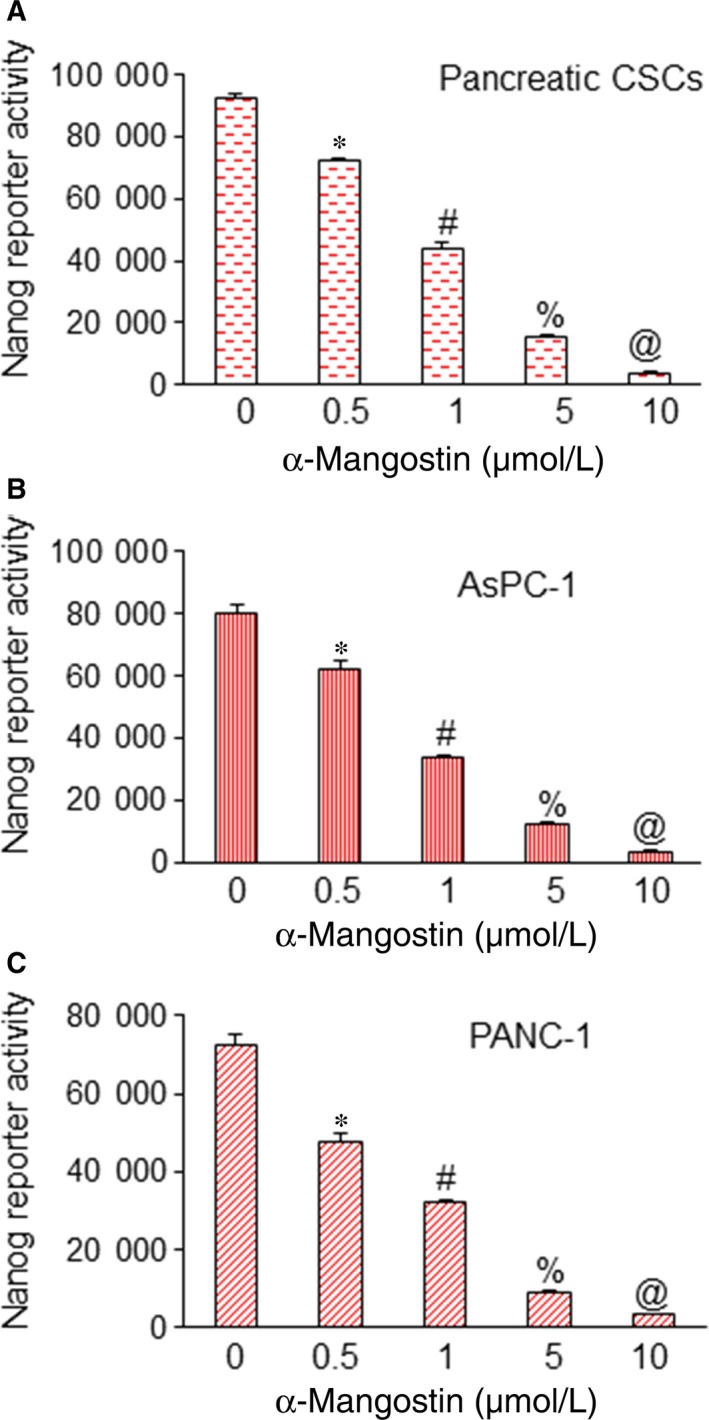
α‐Mangostin inhibits Nanog transcription. (A‐C), Pancreatic CSCs and cancer cell lines (AsPC‐1 and PANC‐1) were transduced with Nanog‐responsive GFP/firefly luciferase viral particles (pGreen Fire1‐Nanog with EF1, System Biosciences). After transduction, the culture medium was replaced, and cells were treated with α‐Mangostin (0‐10 µmol/L) for 24 h. Nanog reporter activity was measured as we described elsewhere^16^ *, #, % and @ = significantly different from control, and each other, *P* < 0.05

As Nanog is also a direct transcriptional target of Gli. To investigate the effects of α‐Mangostin on Nanog transcription, we measured the luciferase reporter activity. In pancreatic CSCs, AsPC‐1 and PANC‐1 cell lines, α‐Mangostin significantly inhibited the Nanog reporter activity (Figure 5). Thus, taken together, these results suggest that α‐Mangostin regulates pluripotency‐, cell survival‐ and cell cycle‐related genes by modulating Cdk2, Cdk6, FGF4, c‐Myc and Oct4 expression through Nanog.

### 3.5 Inhibitory effects of α‐Mangostin on cell motility, migration, invasion and markers of epithelial‐mesenchymal transition

**Figure 6:**
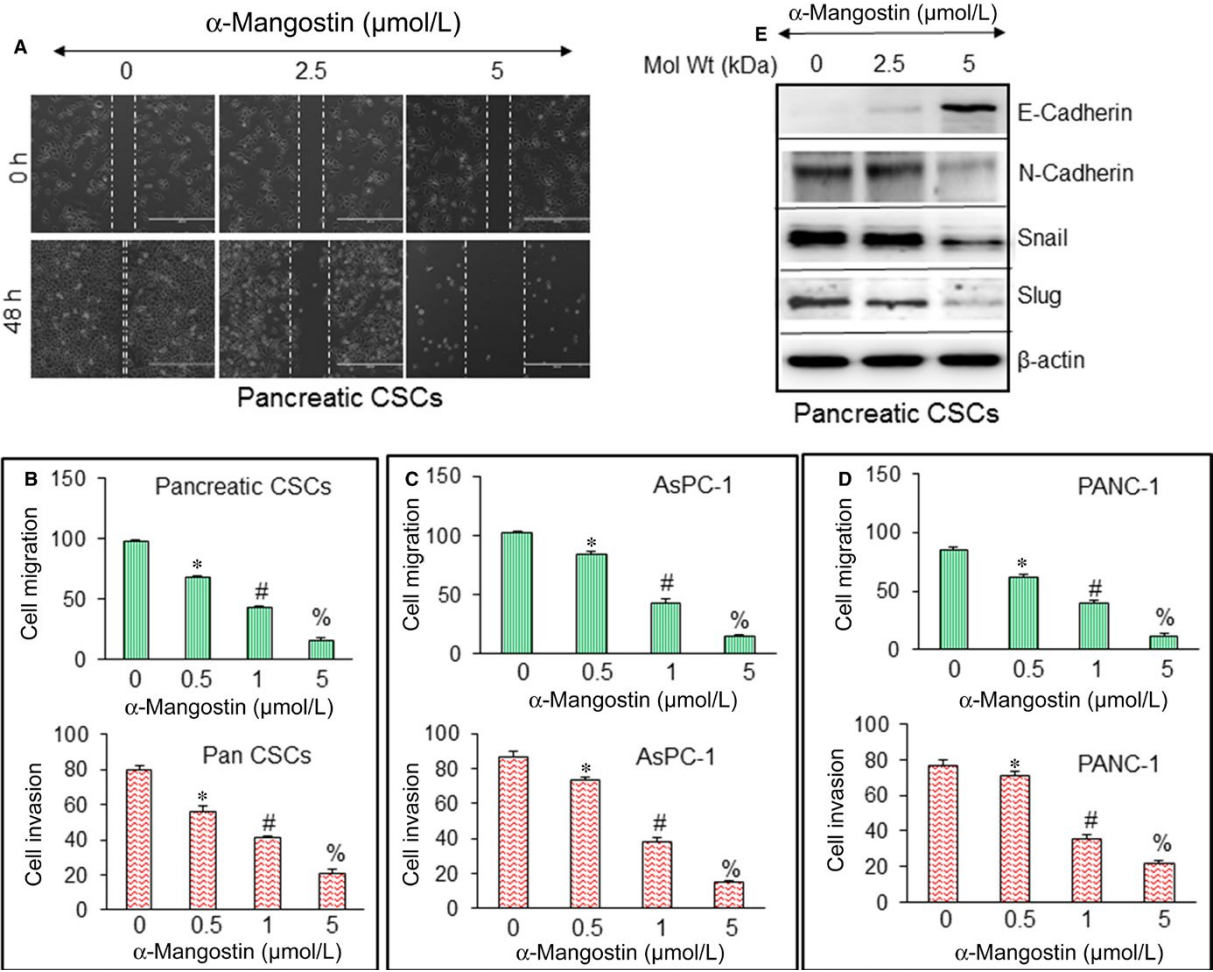
α‐Mangostin inhibits cell motility, migration and invasion and modulates the expression of epithelial‐mesenchymal transition (EMT) markers. A, Pancreatic CSCs isolated from primary tumours were grown in monolayer, scratched and treated with or without α‐Mangostin (0‐5 µmol/L) for 48 h. Cells were photographed as we described elsewhere.^11^, ^13^ (B‐D), Cell Migration and invasion assay. Pancreatic CSCs, AsPC‐1 and PANC‐1 cells were seeded, treated with α‐Mangostin (0‐5 µmol/L) for 48 h and cell migration and invasion assays were performed as described in Material and Methods. Data represent mean (n = 4) ± SD. *, #, % and %= significantly different from control, and each other, *P* < 0.05. (E), Pancreatic CSCs were treated with α‐Mangostin (0‐10 µmol/L) for 48 h. The expression of E‐cadherin, N‐cadherin, Snail and Slug was measured by the Western blot analysis. β‐actin was used as an internal control

For metastasis to occur, EMT becomes inevitable in which cancer cells acquire genetic changes that equip them to migrate to distant organ sites where they can reestablish and proliferate.^34^, ^35^ As CSCs are associated with the metastasis and treatment resistance, we further examined the effects of α‐Mangostin on acquiring metastatic characteristic namely cell motility, migration, invasion and expression of EMT markers. Figure 6A,B demonstrate that α‐Mangostin inhibits cell motility, migration and invasion of pancreatic CSCs. Further as shown in Figure 6C,D α‐Mangostin showed similar inhibitory effects on cell migration and invasion of AsPC‐1 and PANC‐1 cell lines.

α‐Mangostin inhibited cell motility, migration and invasion, using Western blot assay therefore we next examined the effects of α‐Mangostin on the expression of proteins, involved in the regulation of EMT. Transcription factors Snail and Slug regulate the expression of cadherins. As demonstrated in Figure 6E, α‐Mangostin induces the expression of E‐cadherin while inhibiting the expression of N‐Cadherin, and transcription factors Snail and Slug. Thus, suggesting that α‐Mangostin has the potential to inhibit EMT.

### 3.6 Hyper‐activation of Sonic hedgehog pathway or overexpression of Nanog counteracts the inhibitory effects of α‐Mangostin

**Figure 7:**
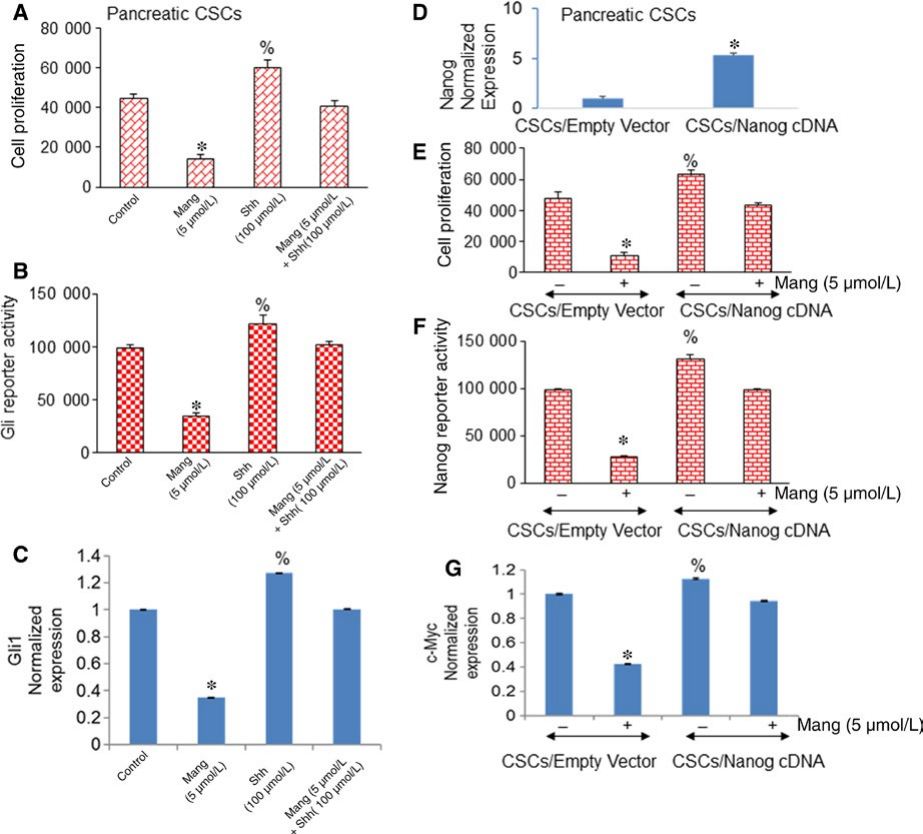
Enforced activation of the Shh pathway or Nanog overexpression abrogated inhibitory effects of α‐Mangostin on cell proliferation and Gli or Nanog transcription and expression respectively. A, Pancreatic CSCs were pretreated with Shh protein (100 nmol/L) for 2 h followed by treatment with α‐Mangostin (5 µmol/L) for 48 hrs. Cell proliferation was measured by trypan blue assay. Data represent mean (n = 4) ± SD. *, or % = significantly different from control, *P* < 0.05. B, Using Gli‐responsive GFP/firefly luciferase viral particles (pGreen Fire1‐Gli with EF1, System Biosciences), pancreatic CSCs were transduced. After transduction, CSCs were pretreated with Shh protein for 2 h followed by treatment with α‐Mangostin for 24 h. Gli transcription was measured by luciferase assay as we described elsewhere.^15^ Data represent mean (n = 4) ± SD. *, or % = significantly different from control, *P* < 0.05. C, Pancreatic CSCs were pretreated with Shh protein (100 nmol/L) for 2 h followed by treatment with α‐Mangostin (5 µmol/L) for 48 h. The expression of Gli1 was measured by q‐RT‐PCR. Data represent mean (n = 4) ± SD. *, or % = significantly different from control, *P* < 0.05. D, Pancreatic CSCs were transduced with lentiviral particles expressing either empty vector or Nanog cDNA. The expression of Nanog was measured by q‐RT‐PCR. Data represent mean (n = 4) ± SD. * = significantly different from control, *P* < 0.05. E, Pancreatic CSCs were transduced with lentiviral particles containing either empty vector or Nanog cDNA and treated with Mang (5 µmol/L) for 48 h. Cell proliferation was measured by trypan blue assay. F, Pancreatic CSCs (CSCs/Empty vector and CSCs/Nanog cDNA) were transduced with Nanog‐responsive GFP/firefly luciferase viral particles (pGreen Fire1‐Nanog with EF1). After transduction, CSCs were treated with Mang for 24 h. Nanog transcription was measured by luciferase assay as we described elsewhere.^15^ Data represent mean (n = 4) ± SD. *, or % = significantly different from control, *P* < 0.05. G, CSCs/Empty Vector and CSCs/Nanog cDNA cells were treated with Mang (5 μmol/L) for 48 h, and c‐Myc expression was measured by q‐RT‐PCR. Mang = α‐Mangostin

As α‐Mangostin inhibited the transcription and expression of Gli, to counteract the inhibitory effects of α‐Mangostin on cell proliferation, Gli reporter activity and Gli1 expression, we examined whether hyper‐activation of Shh pathway by Shh protein can abrogate this phenotype. As shown in (Figure 7A‐C), α‐Mangostin inhibited cell proliferation, Gli transcription and expression. Incubation of CSCs in the presence of Shh protein slightly enhanced the cell proliferation, Gli transcription and expression. Furthermore, Shh protein abrogated the inhibitory effects of α‐Mangostin on cell proliferation, and Gli transcription and expression. Altogether, these data suggest that inhibition of the self‐renewal capacity of pancreatic CSCs by α‐Mangostin is in part due to the suppression of the Shh pathway.

As Nanog is also a transcriptional target of Gli, we further examined whether by overexpressing Nanog the inhibitory effects of α‐Mangostin on pancreatic CSC proliferation, Nanog transcription and its target c‐Myc can be abrogated. Thus, lentiviral particles expressing either empty vector or Nanog cDNA were used to transduce the pancreatic CSCs. Overexpression of Nanog in CSCs (CSCs/Nanog cDNA) significantly increased the Nanog expression as compared with that in CSCs/Empty vector group (Figure 7D). Cell proliferation and Nanog transcription and expression were measured in the transduced pancreatic CSCs treated with α‐Mangostin. α‐Mangostin inhibited cell proliferation, and Nanog transcription and expression in pancreatic CSCs/Empty vector group (Figure 7E‐G). However, these inhibitory effects of α‐Mangostin in pancreatic CSC/Nanog cDNA group were counteracted by the overexpression of Nanog. We can thus conclude that the biological effects α‐Mangostin are exerted through the inhibition of Shh‐Nanog pathway, which can regulate the pluripotency and self‐renewal capacity of pancreatic CSCs.

## 4 DISCUSSION

In the current study, we demonstrate that α‐Mangostin inhibits pancreatic CSC characteristics through Shh‐Nanog pathway. Specifically, α‐Mangostin inhibits markers of pancreatic CSCs, pluripotency maintaining factors and Nanog transcription. α‐Mangostin inhibits cell motility and markers of EMT in CSCs and may act as a Gli transcription inhibitor. The inhibitory effects of α‐Mangostin on pancreatic CSC proliferation and Gli or Nanog transcription and their targets were abrogated by either enforced activation of Shh or by the overexpression of Nanog.

We and others have documented the biological significance of pancreatic CSCs. α‐Mangostin, a non‐toxic xanthonoid, inhibits the cell proliferation in pancreatic CSCs and cancer cell lines. Also, α‐Mangostin inhibits the colony formation and induces apoptosis in these cells as well, compared to the pancreatic normal ductal epithelial cells. Several recent studies have demonstrated the roles of CSCs in malignant transformation, cancer initiation, metastasis, recurrence and drug resistance. The ability of α‐Mangostin to inhibit pancreatic CSC markers CD24, CD44 and CD133 demonstrate that it can modulate the tumour growth by the suppression of the CSC population. Furthermore, the ability of α‐Mangostin to suppress the expression of pluripotency maintaining genes Oct4, Sox2, KLF4 and c‐Myc suggest that it can directly control cancer stem cells, proliferation, pluripotency and self‐renewal.

Sonic hedgehog pathway in pancreatic cancer has been shown to be constitutively active and plays a significant role in the initiation, progression and metastasis.^3^ Gli binds to GACCACCCA motif and regulates the transcription of Gli1, Patched1, Patched2, HHIP1, Myc‐N, Cyclin D1, Cyclin D2, Bcl‐2, JAG2, GREM1, CFLAR, FoXF1, FoXL1, Follistatin and PRDM1. HH signals are fine‐tuned based on positive feedback loop via Gli1 and negative feedback loop via Patched and HHIP1. We have shown that several natural products can regulate pancreatic CSC characteristics and inhibit tumour growth by suppressing the Shh‐Gli pathway.^8^, ^9^, ^10^, ^11^, ^32^, ^36^, ^37^, ^38^, ^39^ In the present study, we demonstrate that α‐Mangostin modulates several critical components of the Shh pathway to inhibit the proliferation of pancreatic cancer cell lines and CSCs. α‐Mangostin inhibited direct (Gli and Patched) as well as indirect (Smoothened) targets of Gli. We are unable to provide reasons for inhibition of smoothened by α‐Mangostin. It is possible that α‐Mangostin, like other natural products, inhibits different transcription factors which in turn can target smoothened. Lei et al have demonstrated that α‐Mangostin regulates invasion of pancreatic cancer cells and suppresses PSC activation induced by hypoxia through inhibiting the stabilization of HIF‐1α and Gli1 expression.^30^ Overall, these data together suggest that α‐Mangostin may be beneficial for inhibiting pancreatic CSC characteristics and also by preventing pancreatic cancer progression induced by hypoxia.

Nanog has been shown to play an essential role in the maintenance of self‐renewal capacity and pluripotency in embryonic stem cells and progenitor cells.^20^ Further, it has been shown that Nanog is expressed in multiple tumour types including pancreatic cancer and is associated with poor patient survival. Nanog enhances molecular events required for tumour progression and thus represents as a potential biomarker and a plausible therapeutic target. We have previously demonstrated that Nanog may act as an oncogene and thus promotes carcinogenesis. Silencing of Nanog by shRNA inhibited tumour growth and pancreatic CSC characteristics. Thus, Nanog expression may be involved in the cell fate determination of pluripotent CSCs. Shh pathway can regulate the expression of Nanog. Here, we have shown using ChIP assay that Nanog directly binds to Cdk2, Cdk6, FGF4, c‐Myc and Oct4 promoters, and that α‐Mangostin treatment can abrogate the binding of Nanog to these gene promoters. Overexpression of Nanog rescued α‐Mangostin‐induced cell death. Our data suggest that by targeting Nanog, α‐Mangostin regulates the cell cycle, pluripotency and self‐renewal of pancreatic CSCs.

Inhibition of Shh pathway at the level of Gli transcription will be more effective in treating patients because mutations in smoothened receptors (upstream of Gli) are commonly observed during treatment. Our studies define the new mechanism(s) and novel molecular targets of pancreatic cancer treatment and prevention. α‐Mangostin can regulate progression and metastasis through the inhibition of Shh signalling pathway and factors controlling pluripotency and EMT. In the present study, we have used α‐Mangostin doses up to 10 µmol/L, which are comparable to other chemodietary agents in the clinic. This dose is low enough to exert clinical impact and may not need any further modification. In another study, we have demonstrated the pre‐clinical potential of α‐Mangostin‐encapsulated PLGA nanoparticles for the management of pancreatic cancer.^40^ Thus, α‐Mangostin offers excellent potential as a novel preventive and therapeutic agent for pancreatic cancer by targeting CSCs.

Both EMT and CSCs have been increasingly demonstrated to be associated with the development of invasive characteristic and distant metastasis.^41^, ^42^ CSCs have also been shown to express genes associated with EMT along with stemness genes. HH promotes EMT by upregulating the expression of Snail, Slug, ZEB1, ZEB2, TWIST2 and FOXC2. Snail inhibits cadherin gene expression and triggers EMT. Snail has been shown to bind to the E‐cadherin promoter at the E‐box site and thus can repress the transcription of E‐cadherin transcription.^43^ Further, it has been reported that Snail also stimulates the transcription of mesenchymal genes such as N‐cadherin and Vimentin and thus regulate their expressions.^44^

In conclusion, we demonstrate here that α‐Mangostin inhibits pancreatic CSC characteristics and cancer cell growth through the inhibition of Shh‐Gli pathway. α‐Mangostin inhibits Gli transcription, and its target genes Nanog, Oct4, c‐Myc, Sox2 and KLF4. α‐Mangostin not only inhibited smoothened but also Gli transcription. Therefore, α‐Mangostin has an additional clinical advantage, that is, if the pancreatic cancer patients develop resistance to smoothened inhibition due to mutation(s) on the smoothened receptors, they will be able to respond to α‐Mangostin because it also inhibits the expression of effector molecule Gli. Therefore, targeting the Shh‐Gli pathway through dual inhibition (smoothened and Gli) by α‐Mangostin could have enormous clinical significance for pancreatic cancer initiation, progression, metastasis and tumour growth. Overall, our study suggests that α‐Mangostin can be used for the treatment and prevention of pancreatic cancer by targeting CSCs.

## CONFLICT OF INTEREST

YM, W.Y and AS have declared that no competing interests exist. SS and RKS have declared intellectual property interest.

## AUTHOR CONTRIBUTIONS

WY and YM performed the experiments, analysed the data and wrote the manuscript; SS, AS and RKS designed the study and contributed reagents. All authors read and approved the manuscript.
